# Insulin: The Friend and the Foe in the Development of Type 2 Diabetes Mellitus

**DOI:** 10.3390/ijms21051770

**Published:** 2020-03-05

**Authors:** Nadia Rachdaoui

**Affiliations:** Department of Animal Sciences, Room 108, Foran Hall, Rutgers, the State University of New Jersey, 59 Dudley Rd, New Brunswick, NJ 08901, USA; rachdaoui@sebs.rutgers.edu; Tel.: +1-848-932-6320

**Keywords:** insulin, autocrine, pancreatic β-cell, hyperinsulinemia, β-cell mass, compensation, decompensation and type 2 diabetes

## Abstract

Insulin, a hormone produced by pancreatic β-cells, has a primary function of maintaining glucose homeostasis. Deficiencies in β-cell insulin secretion result in the development of type 1 and type 2 diabetes, metabolic disorders characterized by high levels of blood glucose. Type 2 diabetes mellitus (T2DM) is characterized by the presence of peripheral insulin resistance in tissues such as skeletal muscle, adipose tissue and liver and develops when β-cells fail to compensate for the peripheral insulin resistance. Insulin resistance triggers a rise in insulin demand and leads to β-cell compensation by increasing both β-cell mass and insulin secretion and leads to the development of hyperinsulinemia. In a vicious cycle, hyperinsulinemia exacerbates the metabolic dysregulations that lead to β-cell failure and the development of T2DM. Insulin and IGF-1 signaling pathways play critical roles in maintaining the differentiated phenotype of β-cells. The autocrine actions of secreted insulin on β-cells is still controversial; work by us and others has shown positive and negative actions by insulin on β-cells. We discuss findings that support the concept of an autocrine action of secreted insulin on β-cells. The hypothesis of whether, during the development of T2DM, secreted insulin initially acts as a friend and contributes to β-cell compensation and then, at a later stage, becomes a foe and contributes to β-cell decompensation will be discussed.

## 1. Introduction

Diabetes mellitus (DM), a chronic disease characterized by impaired glucose homeostasis, is a rising epidemic worldwide. The World Health Organization (WHO) reported that, globally, more than 425 million people are living with diabetes, and in the United States alone, more than 30 million Americans have diabetes. The prevalence of DM nearly doubled between 1980 and 2014, rising from 4.7% to 8.5%, and is expected to increase another 50% by 2045. According to the WHO, DM was the direct cause of 1.6 million deaths making it the seventh leading cause of death in 2016. DM patients are at high risk of developing chronic comorbidities and secondary complications, such as neuropathy, retinopathy, nephropathy and cardiovascular disease, leading to a substantial economic burden of $327 billion according to estimates by the American Diabetes Association [1].

DM is a metabolic disorder characterized by high levels of blood glucose or hyperglycemia. Deficiencies in insulin secretion by pancreatic β-cells or in insulin sensitivity and use by peripheral tissues result in the development of DM (i.e., Type 1 and Type 2) [2]. The vast majority (95%) of patients with diabetes have Type 2 diabetes mellitus (T2DM) and are mostly adults (20–79 years old), although in recent years, the incidence of T2DM in youth (20 years and younger) has been on an alarming rise and is thought to be mainly due to a dramatic increase in the rate of childhood obesity. In fact, obesity (defined as a body mass index of greater than 30 kg/m^2^) is a major independent risk factor for the development of T2DM [2,3,4]. In the US alone, 35.7 % of adults are obese, and approximately 17% of children (12.5 million), between the ages of 2 and 19, suffer from obesity. Genetic predispositions that result in altered energy homeostasis have been suggested as factors in the development of obesity [5]. However, the current belief is that changes in modern lifestyles, i.e., with abundant nutrients [6] and physical inactivity [7], have resulted in the recent alarming rise in the rates of obesity and associated chronic metabolic diseases, such as T2DM, metabolic syndrome, high blood pressure and dyslipidemia. Although Type 1 diabetes is not brought on by obesity, people with Type 1 diabetes can develop obesity and insulin resistance as a result of long term insulin therapy, which could require combination therapies for treatment [8].

Obesity is characterized by insulin resistance in peripheral tissues, such as skeletal muscle, adipose tissue and liver. Peripheral insulin resistance leads to a rise in insulin demand and triggers pancreatic β-cell adaptation by increasing both β-cell mass and function to release sufficient insulin and therein maintain normoglycemia. This compensatory response results in insulin hypersecretion and the development of hyperinsulinemia; in a vicious cycle, insulin circulates at levels higher than normal and therein participates in the metabolic dysregulations observed in obesity and T2DM [9,10]. Persistent hyperinsulinemia contributes to peripheral insulin resistance by a variety of mechanisms, including decreasing insulin receptor expression and altering intracellular signaling cascades, i.e., inhibition of insulin receptor (IR) kinase activity [11,12] and insulin receptor substrates-1 and -2 (IRS_1/2_) tyrosine phosphorylation, increasing IRS_1/2_ proteasome-mediated degradation [13,14], phosphatase-mediated dephosphorylation and kinase-mediated serine/threonine phosphorylation of IRS_1/2_ [15,16].

Insulin resistance and hyperinsulinemia precede the development of hyperglycemia, the latter develops only when β-cells fail to compensate for peripheral insulin resistance. A myriad of factors, such as cytokines, free fatty acids and hyperglycemia, were suggested as mediators of β-cell decompensation during the development of T2DM. Mitochondrial coupling of β-cell glucose metabolism to insulin secretion was also shown to be impaired in T2DM and suggested as a contributing factor to β-cell decompensation [17,18]. Although insulin signaling is known to be essential to β-cell growth and function [19,20,21], possible long term negative autocrine actions of insulin (i.e., hyperinsulinemia) on β-cell function and mass are still controversial. Work by our group and by others have reported such long term actions of insulin on β-cells [22,23,24]. The goal in undertaking this review was to summarize evidence that supports the principal idea of an autocrine action of insulin on β-cells. We will discuss both the positive and negative autocrine actions of insulin on β-cell function and mass. Interactions between the insulin and insulin-like growth factor-1 (IGF-1) signaling pathways will be addressed in this context, as these pathways play critical roles in β-cell heath. In addition, we will present a conceptual model that could explain positive and negative actions of insulin, in which insulin initially acts as a friend by promoting β-cell compensation during the insulin-resistant prediabetic stage but at later stages insulin becomes a foe by contributing to β-cell decompensation during the development of T2D.

## 2. Insulin and Insulin Signaling

Insulin is a 51 amino acid dipeptide containing an A chain and a B chain linked by 2 disulfide bonds derived from cysteine residues. The A chain has 21 amino acids and the B chain 30 amino acids. Insulin is encoded by the short arm of chromosome 11 in pancreatic β-cells as 100 amino acids (referred to as pre-proinsulin) which comprises a signal peptide, the B chain, a connecting (C) peptide and the A chain [25,26]. Removal of the signal peptide forms proinsulin. In proinsulin, the connecting C-peptide flanked at each end by dibasic residues (Arg-Arg and Lys-Arg) links the N-terminus of the A chain to the C-terminus of the B chain [26,27]. Proinsulin in vesicles formed from the Golgi apparatus is converted to insulin by removal of the dibasic residues by trypsin-like endoprotease enzymes to insulin and C-peptide [26]. Insulin, in mature secretory granules, is stored as hexameric unites coordinated by 2 axial zinc (Zn^2+^) ions in the center of the hexamer [26]. These insulin hexamers are hydrophobic and stable at a pH of 5–5.5 in β-cell secretory granules [26,27]. It is unlikely that a substantial amount of these insulin hexamers exist in the plasma, especially that Zn^2+^ concentration in plasma is low [26]. Insulin and C-peptide are secreted by β-cells into the circulation by exocytosis in an equimolar ratio, with very small amounts of proinsulin (2–3%) [27].

It is the insulin monomer that binds to receptors and triggers downstream cascades of signal transduction to mediate various cellular functions [28,29]. Insulin receptors belong to the superfamily of receptor tyrosine kinases, characterized by 2 halves comprised each of an extracellular portion that specifically binds insulin, and a transmembrane portion with an intracellular tyrosine kinase domain [30]. Each half of the insulin receptor is composed of an N-terminal alpha chain and a C-terminal beta chain (i.e., α- and β-subunits) linked by a single disulfide bond. The α-subunit is the extracellular domain that binds insulin, whereas the β-subunit has the transmembrane domain and the intracellular tyrosine-kinase activity domain [28,29]. The insulin pro-receptor is encoded by a 150 Kb gene on chromosome 19 with 22 exons and 21 introns [31]. Two receptor isoforms, insulin receptor-A and -B (IR-A and IR-B), that differ slightly in their affinity for insulin, are encoded by alternative splicing of exon 11, which encodes a 12-amino acid sequence in the C-terminus of the α-subunit of the receptor [31]. IR-A lacks exon 11, while IR-B contains it [32,33].

**Figure 1:**
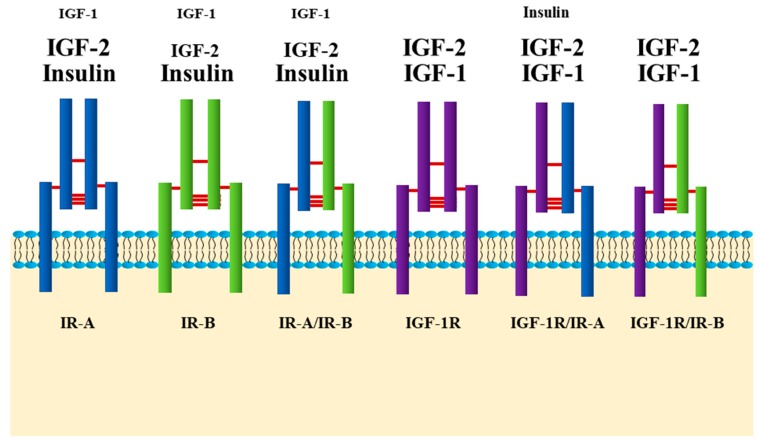
Insulin and insulin-like growth factor (IGF)-1 receptors. There are six types of receptors: insulin receptor (IR)-A, IR-B, IGF-1R and hybrid receptors, IR-A/IR-B, IGF-1R/IR-A and IGF-1R/IR-B. Expression of IR-A and IR-B is tissue specific. In adult life, IR-B is predominantly expressed in insulin target tissues such as liver, muscle and adipose tissue. IR-A is predominantly expressed in fetal tissues, and its expression declines during adulthood. IR-A and IR-B receptors can form hybrid receptors with each other, or along with one half of the IGF-1R. These receptors, the IGF-1R and hybrids bind the three ligands, insulin, IGF-1 and IGF-2 with different affinities. This figure depicts the order of their affinities to the different ligands by the size of the ligand font. Smaller font indicates lower affinity, and bigger font, higher affinity.

Researchers often tend to compartmentalize their work by categorizing insulin signaling as “metabolic” and insulin-like growth factor-1 (IGF-1) signaling as “growth promoting”. It is probably more accurate to consider this receptor family in a more unified fashion. The insulin and IGF-1 receptors are highly homologous and share a myriad of overlapping downstream signaling molecules, with very subtle differences. Insulin and IGF-1 receptors, in a similar fashion, are synthesized as single chain pre-proreceptors that are cleaved and processed to yield dimerized mature A and B receptors. Unlike other receptor tyrosine kinases, the IR and IGF-1 receptors are covalently disulfide-linked dimers with two extracellular α-subunits and two transmembrane β-subunits [29,34]. Although they are relatively specific for their respective ligands, the IR-B isoform binds IGF-1 with ~100 times lower affinity than insulin, while the IR-A isoform has a higher affinity for IGF-1, and during embryonic development for IGF-2 that is close to that for insulin [35]. On the other hand, IGF-1R binds insulin with a ~100 times lower affinity than its cognate ligand, IGF-1, while the so-called hybrid receptors formed between IR and IGF-1R have a much higher affinity for insulin (Figure 1) [36,37,38].

In cells expressing both insulin and IGF-1 receptors, hybrid receptors, consisting of one half of each, are formed [39]. Tissues, such as adipose and liver tissue, that express the insulin receptor (specifically IR-B) more abundantly than the IGF-1 receptor, have lower proportions of hybrid receptors (i.e., 17% to 45%) and are considered classic insulin-responsive tissues [40,41]. Others, such as pancreas and brain, that equally express insulin and IGF-1 receptors have higher proportions of hybrid receptors (up to 60%) [41], although there are cell type specific differences in the expression of the IRs and IGF-1R in these heterogenous tissues [42,43,44]. Hybrid receptors composed of αβ subunits from the IR-B and the IGF-1R selectively bind IGF-1, whereas hybrid receptors composed of αβ subunits from the IR-A and the IGF-1R bind insulin and the IGFs with similar affinities (Figure 1) [35]. The physiological roles of insulin/IGF-1 hybrid receptors in cell function are still not well understood; they were suggested to enhance cell responsiveness to the IGFs [35].

Insulin binding to both α-subunits of the IR exhibits negative cooperativity as shown by the curvilinear Scatchard plot and the accelerated dissociation of the pre-bound insulin in the presence of free insulin ligand [45,46], indicating the coexistence of 2 distinct, but equivalent, insulin binding sites with low and high affinity on the IR (referred to as Site 1 and Site 2, respectively) [45,47,48]. This phenomenon is dependent on the dimeric nature of the insulin receptor [49]. One insulin molecule binds to the receptor dimer with high affinity, and other insulin molecules bind with lower affinity due to a ligand-induced receptor asymmetry [45]. Insulin binding to the receptor dimer is speculated to induce conformational changes that result in the proximation of the intracellular tyrosine kinase domains, their tri-phosphorylation and activation, and the subsequent transphosphorylation of tyrosine residues outside the kinase domain on the β-subunits. This creates docking sites for signaling protein containing phosphotyrosine-binding domains (PTB) and Pleckstrin domains, such as the insulin receptor substrate proteins, IRS_1-6_ [45,50,51]. IRS_1-4_ proteins are well characterized. When recruited to the activated receptor, IRS proteins, undergo phosphorylation on multiple tyrosine residues that form binding sites for downstream signaling molecules that contain Src-homology 2 domains (SH2) [50,51]. The two main downstream signaling pathways of insulin signaling activated by the IR-IRS interaction are the phosphatidylinositol 3-kinase (PI3K)/protein kinase B (PKB, also known as AKT) signaling pathway [51,52] and the Raf/Ras/MEK/ERK (extracellular signal regulated kinase, also known as mitogen activated protein kinase, MAPK) pathway [51]. Insulin activation of the PI3K/PKB pathway is known to mostly control metabolic functions, while activation of the Raf/Ras/MEK/ERK pathway through IRS and Shc, regulates cell growth and differentiation [53].

## 3. Insulin Secretion and Signaling in β-Cells

The function of insulin is to maintain blood glucose levels in a homeostatic range by stimulation of glucose uptake in insulin target tissues, such as skeletal muscle and adipose tissue, and inhibition of glucose output by the liver. Glucose is the primary regulator of insulin secretion by pancreatic β-cells, triggering a cascade of events referred to as stimulus-response coupling. β-cells have the capacity to sense circulating glucose levels and secrete the appropriate amount of insulin to keep blood glucose in a normal range. As circulating glucose levels rise (> 8–10 mM), for example after a meal, glucose is transported into the β-cell through the glucose transporter GLUT2 by facilitated diffusion. GLUT2 is the only glucose transporter expressed in β-cells and has low substrate affinity ensuring high glucose influx. After entering the β-cell, glucose is phosphorylated by the rate-limiting enzyme glucokinase. Glucokinase functions as a glucose sensor in β-cells; it has a lower affinity for glucose than other hexokinases (Km ~ 6 mM/L) and is not inhibited by its product [54,55,56,57]. Glucose is then metabolized through glycolysis and the tricarboxylic acid cycle (TCA cycle, also known as Krebs cycle) to generate ATP and increase the ATP/ADP ratio. This causes closure of the ATP-dependent K+-channels, depolarization of the plasma membrane and activation of the voltage-dependent Ca^2+^-channels. Ca^2+^ influx and the increase in intracellular Ca^2+^ concentration then leads to insulin containing exocytotic granules to dock to the plasma membrane and release insulin hexamers [56,57].

Due to the important role that insulin plays in maintaining glucose homeostasis, it was suggested that the processes of its secretion and biosynthesis are tightly controlled in β-cells. Glucose is the major physiological regulator of insulin gene transcription and mRNA translation [58,59]; a commonly accepted concept is that glucose exerts its immediate effect through a feed-forward loop via secreted insulin activation of IR and downstream signaling pathways. Work by Efrat et al. (1991) [60] and Leibiger et al. (1998 and 2000) [61,62] confirmed this concept using nuclear run-off experiments. They demonstrated that up-regulation of insulin biosynthesis occurred within minutes of glucose stimulation, suggesting a positive autocrine feedback loop in which secreted insulin enhances its own biosynthesis. These studies were important in delineating the mechanisms underlying the short-term glucose-mediated control of insulin gene transcription. This key finding, that secreted insulin, in response to glucose stimulation, controls its own biosynthesis [61], paved the way for other researchers, including our group, to study the autocrine actions of insulin on the same cells that produce it, β-cells of the endocrine pancreas.

There are two opposing schools of thought regarding this concept. The first view is that β-cells are constantly exposed to high concentrations of insulin due to continuous insulin secretion, even at basal levels of glucose concentrations (< 5 mM); therefore, they must be irresponsive to the actions of insulin as a result of the desensitization of insulin receptors and downstream signal transduction pathways. This argument contradicts the key observation that, as in other insulin target cells, IR desensitization is often associated with internalization and down-regulation of receptor number at the cell plasma membrane [63,64,65]. Therefore, according to this argument, if β-cells are continuously exposed to high concentrations of insulin, even in physiological conditions, IRs in β-cells should perhaps be inexistent. However, studies using single-cell reverse transcription PCR or ^125^I-labeled insulin have shown that islet β-cells express all subtypes of IRs, as well as all downstream signaling components [19,66,67,68,69,70,71,72,73], suggesting that these receptors and their downstream signaling elements have significant purposes in β-cells.

This basic notion of secreted insulin directly acting on its IRs on β-cells is still widely debated, although in the last 40 years, extensive research using global and conditional knockout mouse models of different components of the insulin signaling pathway (i.e., IR and IRS proteins), as well as isolated rodent and human islets and different β-cell lines, have clearly confirmed the critical contributions of this pathway in maintaining β-cell function and mass [74,75,76,77,78,79]. One of the major arguments that reinforces this disagreement are reports which demonstrate opposing findings of negative and positive actions of insulin on β-cell function and survival; therefore, a consensus on the autocrine actions of insulin on β-cells has not yet been reached.

Our view on this concept, which belongs to a second school of thought, is that, like any other insulin target cells in the body, since pancreatic β-cells possess all of the necessary signaling components of the insulin signaling pathway, they must have the ability to respond (via autocrine actions) to secreted insulin. We further argue that the positive and negative actions of insulin, that were reported by researchers, are two sides of the same coin and might reflect both physiological and pathological actions of insulin on β-cells. We suggest that these positive and negative actions of insulin may depend on the β -cell’s surrounding microenvironment. In other words, responsiveness of β-cells to secreted insulin might be dependent on 1) changes in the insulin concentration around β-cells, i.e., normal or high, 2) the duration of exposure, i.e., acute versus prolonged exposure of β-cells to insulin and 3) whether glucose is present at basal or high concentrations around β-cells. We will discuss our view and provide evidence that supports this “unifying concept” on the positive and negative autocrine actions of insulin on β-cells.

## 4. Insulin as a Friend: Positive Autocrine Actions

Positive autocrine actions of insulin through binding to its surface receptors on β-cells have been reported and seem to affect multiple aspects of β-cell function including (i) regulation of glucose-mediated insulin secretion, (ii) control of insulin gene expression, (iii) inhibition of apoptotic β-cell death and (iv) promotion of β-cell survival, differentiation and proliferation. We will next outline these different aspects of the positive autocrine actions of insulin and discuss recent findings in the literature which suggest that insulin signaling is essential to β-cell function and mass.

### 4.1. Positive Actions of Insulin on Insulin Gene Expression and Insulin Secretion

Recent work has suggested that secreted insulin, by an autocrine feed-forward loop, directly acts on its receptors in β-cells and enhances its own production. The release of insulin from readily available pools in response to glucose immediately activates translation and transcription of the insulin gene to replenish insulin pools [61,62,73,80,81,82]. The order of events in response to the rise of glucose was shown to be compatible with this concept, particularly that insulin secretion is triggered within seconds to minutes after the addition of glucose, translation of pre-proinsulin mRNA within minutes and insulin gene transcription within ~ 1 h [61,83]. 

This short-term regulation by secreted insulin of its own gene expression is mediated through the IR-A, IRS_2_, Class IA PI3K (PI3KIa), p70S6k, Ca2+/calmodulin-dependent kinase II (CaMKII) pathway [80]. A study by Xu and Rothenberg (1998), using βTC6-F7 β-cells overexpressing either a wild type or a mutant tyrosine kinase inactive (A/K1018) IR, showed that a proportionate increase in tyrosine kinase activity in wild type cells, in presence of stimulatory concentrations of glucose, correlated with an increase in insulin content due to increased insulin mRNA expression, while no change was observed in cells overexpressing mutant tyrosine kinase inactive IRs [73]. These studies clearly suggest that IRs mediate the stimulatory effects of glucose on insulin gene transcription. Furthermore, Leibiger et al. (2000) demonstrated that within 30 min following glucose-mediated stimulation of insulin secretion 50% of the synthesized proinsulin resulted from insulin-induced gene transcription, and the other 50% of pro-insulin was due to glucose-mediated posttranscriptional/posttranslational changes [62]. Therefore, insulin secreted from β-cells in response to glucose induces a positive feed-forward loop to enhance its production through activation of its gene transcription; this constitutes an important physiological mechanism through which insulin demand in response to a rise in glucose concentration is rapidly satisfied [84].

These effects by glucose and insulin seem to be mostly mediated through the transcription factor, pancreatic duodenal homeobox-1 (Pdx1), which is mainly expressed in β-cells [85,86]. Glucose and insulin mediate Pdx1 activation through its phosphorylation and translocation to the nucleus, where it binds to regulatory elements in the insulin gene promoter that contains AT rich sequences known as A-boxes (A1-A5). A-boxes bind transcription factors that belong to the homeodomain-containing protein family, such as Pdx1, a major transactivator of the insulin gene (for a detailed review see Melloul 2004). β-cell specific inactivation of the *Pdx1* gene in mice resulted in loss of β-cell phenotype caused by impaired expression of insulin and the glucose transporter, Glut2; these mice developed T2DM with age [87].

What reinforces the controversy around this concept of whether short-term autocrine actions of insulin affect its own secretion are the different experimental outcomes reported by investigators. Early studies observed inhibitory actions of exogenous insulin on insulin secretion [88,89,90,91,92,93], whereas others reported no effects [94,95,96,97,98]; in contrast, recent studies demonstrated that insulin enhances its own secretion following glucose stimulation [75,99,100,101,102,103]. These discrepancies surrounding short term insulin action on insulin secretion might be due to differences in the experimental preparations used in these studies, such as different concentrations and/or incubation times with exogenous insulin and whether stimulatory concentrations of glucose were present or absent in incubation medium. 

Although it is still controversial, a great body of evidence supports the idea of a short term positive autocrine action of secreted insulin on its own exocytosis. A 4 h pre-exposure to exogenous insulin was shown to increase, by ~40%, the endogenous glucose-stimulated insulin secretory response in healthy humans [104]. Aspinwall et al. (1999b), using single cell amperometric measurements of insulin secretion from preloaded β-cell vesicles with charged 5-hydroxytryptamine (5-HT: serotonin), were the first to demonstrate that added insulin triggers immediate insulin exocytosis by increasing [Ca^2+^] i, through Ca^2+^ mobilization from endoplasmic reticulum stores rather than by plasma membrane depolarization and Ca^2+^ efflux [105]. Later studies made similar findings and suggested that the rapid insulin-mediated increase in [Ca^2+^] i and subsequent insulin exocytosis involved the IR/IRS_1_/PI3K signaling pathway [76,102]. In fact, mouse models of global or β-cell specific knockout of different components of the insulin signaling pathway demonstrated that secreted insulin is essential to glucose-stimulated insulin secretion and to normal β-cell function in general. For example, β-cell specific knockout of the IR (βIRKO) [75,101], global knockout of IRS_1_ [78,106] or islet specific deletion of IRS_2_ (PIrs2KO) [107] resulted in defective glucose-stimulated insulin secretion, and mice developed glucose intolerance and diabetes with age.

### 4.2. Positive Actions of Insulin on β-Cell Mass and Survival

It was previously thought that the pancreas is born with all the β-cells that it will ever have; however, recent evidence from numerous studies has revealed that pancreatic β-cells are remarkably dynamic and are able to adapt and modulate their mass in response to a variety of physiological (i.e., pregnancy) and pathophysiological (i.e., obesity) states [108,109]. β-cells are capable of maintaining their size and responding to insulin demand, such as in conditions of insulin resistance, by balancing proliferation, differentiation and apoptosis [109]. Dor et al. (2004) performed direct lineage tracing of β-cells in transgenic mice using the Cre/lox system and demonstrated that the primary mechanism by which new β-cells are formed is self-duplication of terminally differentiated β-cells, rather than neogenesis from progenitor cells [110]. These findings were later confirmed by several other studies [111,112,113]. β-cell mass is maintained through balanced low rates of proliferation and programed cell death (i.e., apoptosis) [109] (Bonner-weir 2000). However, in certain circumstances, such as in T2DM, the rate of β-cell death by apoptosis outweighs the rate of cell replication [109,114,115].

β-cell mass is regulated by a myriad of factors, including nutrients (i.e., glucose) [116,117], hormones (i.e., PRL, GLP1) [118,119,120] and growth factors (i.e., IGF2) [120,121,122], which activate diverse intracellular signaling pathways. Glucose is the major regulator of β-cell growth and mass [123,124,125] and was shown to modulate downstream signaling molecules in the insulin signaling pathway, such as IRS_2_, PKB (Akt), ERK_1/2_ and the mammalian target of rapamycin (mTOR) [78,121,126,127].

It is now well documented that insulin is an essential regulator of β-cell growth and survival [19,20,21,128,129,130]. βIRKO resulted in decreased β-cell proliferation and reduced mass, which was associated with increased β-cell apoptosis [19]. These βIRKO mice had defective β-cell compensatory mechanisms following exposure to a high fat diet, which was associated with reduced insulin-stimulated FoxO1 phosphorylation and nuclear localization, leading to reduced expression of the β-cell specific transcription factor, Pdx-1, involved in the maintenance of β-cell function and mass [19,20]. In addition, it was shown that global knockout of IR leads to neonatal death and the development of glucose intolerance and diabetes [131]; restoration of functional IRs in the brain, liver and β-cells partially rescues these mice from neonatal death and prevents the development of diabetes, suggesting that insulin signaling in other tissues that are considered non-insulin target tissues, such as islet β-cells, is essential to β-cell compensation and glucose homeostasis [131]. Moreover, in addition to insulin’s actions on β-cell replication [20,21,130], insulin directly inhibits apoptosis via activation of anti-apoptotic mechanisms in human and mouse islets, as well as in β-cell lines; this seems to occur via the activation of the Akt /Pdx1 and the Raf-1/Erk_1/2_ insulin signaling cascades [128,132]. Furthermore, the IRS_2_ branch of the insulin signaling pathway was suggested to play an important role in these antiapoptotic effects by insulin. *Irs2* deficient mice displayed increased β-cell apoptosis and developed T2D [77,78,133,134], while upregulation of IRS_2_ in β-cells prevented diabetes [133].

As discussed earlier, although all of these studies strongly support the essential role of insulin and its signaling pathway in regulating β-cell growth and mass, since insulin and IGF-1 receptors are highly homologous and share several overlapping downstream signaling molecules, the role of IGF-1 signaling was explored by numerous investigators as well. IGF-1R conditional knockout in β-cells (βIGF-1KO) resulted in a progressive loss of glucose-stimulated first phase insulin secretion and glucose intolerance, due to aberrant β-cell proliferation and reduced mass by apoptosis [135]. Local expression of IGF-1 in β-cells prevents β-cell apoptosis in streptozotocin-treated mice and in human islets [136,137], as well as in cytokine exposed mouse islets [138]. Three potential mechanisms were shown to underlie the protective effects of IGF-1: 1) an up-regulation of anti-apoptotic proteins (e.g., bcl-2 and bcl-x), 2) a down-regulation of apoptotic proteins (e.g. Bad and Bax) and 3) an inhibition of caspase 9-mediated mitochondrial-dependent β-cell apoptosis, and these effects appeared to be mediated via Akt [137,139]. These findings, however, were questioned when Ueki et al. (2006) demonstrated that in mice carrying either βIR^+/-^/ βIGF-1R^-/-^ or βIR^-/-^/ βIGF-1R^-/-^, insulin-signaling, but not IGF-1 signaling, protected β-cells against loss by apoptosis [140].

## 5. Insulin as a Foe: Negative Autocrine Actions

Negative autocrine actions of insulin on β-cells can be categorized into two types. First, a short-term inhibitory feedback loop by insulin on its own production and secretion, which is physiological in nature, plays a critical role in the control of insulin secretion, allowing β-cells to put the brakes on insulin secretion when blood glucose reaches normal levels and there is no need to secrete more insulin [88,90,91,141]. Second, long term negative actions might occur during states of insulin resistance and increased insulin secretion, or hyperinsulinemia, which are non-physiological. In conditions of insulin resistance, β-cells initially undergo a phase of adaptation, in that they enhance their insulin secretory capacity and increase their size and number to cope with the insulin demand. However, these compensatory mechanisms ultimately lead to high circulating levels of insulin, or hyperinsulinemia, which in a vicious cycle worsens the peripheral insulin resistance and contributes to the metabolic dysregulations associated with diabetes. Hyperinsulinemia, induced by β-cell compensation, can persist for years before glucose intolerance develops, as a result of β-cell failure and decompensation. Several factors such as glucotoxicity, lipotoxicity and cytokines, have been shown to contribute to β-cell decompensation [142,143,144]. However, the role of long-term exposure to high insulin (i.e., hyperinsulinemia) on β-cell function and mass is still unclear and very controversial. Studies, including ours, have suggested that prolonged exposure to hyperinsulinemia might induce common molecular defects in the insulin/IGF-1 signaling pathway leading to both peripheral and β-cell insulin resistance and therefore contribute to the development of T2DM [22,23,24].

We now have a clear idea on the essential roles that IRs and IGF-1Rs play in β-cell function and mass. Defects in signaling by these receptors were shown to compromise both glucose stimulated insulin secretion and β-cell growth and survival. IRs and IGF-1Rs share several overlapping downstream signaling molecules, and although they are relatively specific for their respective ligands, homologous and heterologous desensitization occurs as hormone concentrations increase [145,146]. In addition, it was shown that desensitization in peripheral cells can occur even at low hormone concentrations; pretreatment of fibroblast cells with physiological concentrations of insulin, as low as 0.1 nM for 48 h, rendered cells refractory to subsequent IGF-1 stimulation [147]. In addition, we should not overlook the role that IR/IGF-1R hybrids might play in these negative actions by prolonged exposure to insulin, especially that these hybrid receptors have a higher affinity for insulin then IGF-1Rs [37,47]. We therefore believe that, in conditions of insulin resistance and hyperinsulinemia, prolonged exposure to high insulin might induce not only β-cell insulin resistance, but also resistance to IGF-1, which could contribute to β-cell failure in T2D. We will further discuss this concept in the next sections and provide evidence from the literature that supports the idea of short-term and long-term negative autocrine actions of insulin on β-cell. The role of IGF-1Rs and IR/IGF-1R hybrids in these negative actions will be discussed. 

### 5.1. Negative Actions of Insulin on Insulin Gene Expression and Insulin Secretion

Negative autocrine actions of insulin on its own secretion and biosynthesis have been studied since the 1960s, when earlier work, using slices of rat pancreas [88], perfused pancreas [90] and isolated islets [89,148], demonstrated that the glucose-stimulated insulin secretion was reduced following the addition of exogenous insulin. These studies suggested a negative feedback loop through which insulin modulates its own secretion. Recent studies confirmed this concept [93,149,150] and proposed that an immediate negative feedback by secreted insulin involved a PI3K-dependent activation of ATP-dependent potassium channels, hyperpolarization of the cell membrane and inhibition of the voltage-dependent Ca^2+^ channels, which led to decreased [Ca^2+^]i and inhibition of insulin secretion [93,150,151]. The insulin/PI3K-mediated opening of KATP channels was shown to be via an increase in PI (3,4,5) *P3* [151,152], which has an oscillatory pattern and seems to depend on local glucose concentrations [153]. In human studies, similar findings were reported in that infusion of insulin, while maintaining a constant plasma glucose level using a glucose-clamp technique, reduced C-peptide release, an indirect surrogate measure of insulin release [92,154].

In an interesting work by Jimenez-Feltstrom et al. (2004), it was suggested that actions by insulin on its own secretion are dose-dependent. Insulin secretion, from isolated mouse islets, was increased at low concentrations of insulin, between 0.05 and 0.1 nM, unchanged at concentrations between 1 and 100 nM and inhibited at concentrations higher than 250 nM [141]. Negative-feedback actions of insulin on its own secretion seem to mostly occur at high concentrations of insulin (ranging from 200 to 1,000 µU/mL) [141,149]. This supports the concept that, initially, insulin acts to enhance its secretion in response to glucose stimulation, however, when local insulin concentration becomes high, secretion is inhibited. Whether these inhibitory actions by secreted insulin are mediated through binding to IR/IGF-1R hybrids and/or IGF-1Rs is unclear. One critical factor that supports this idea is that IGF-1 signaling was shown to inhibit insulin secretion [155,156,157]. Zhao et al. (1997) have shown that IGF-1 inhibits glucose- and glucagon-like peptide 1 (GLP-1)-stimulated insulin secretion. This involved a decrease in 3′,5′-cyclic adenosine monophosphate (cAMP) via activation of the cAMP phosphodiesterase 3B (PDE3B) [157]. It is therefore possible that in presence of high insulin concentrations, the negative actions of insulin are mediated via binding to IR/IGF-1R hybrids and/or IGF-1Rs. We propose that, while the immediate feed forward stimulatory actions of insulin on its own secretion might be mediated via the IRs, at high concentrations of insulin, either during the glucose stimulation of insulin secretion (short-term negative feedback) or during states of insulin resistance and hyperinsulinemia (long-term negative actions), negative actions of insulin might be mediated via insulin binding to IR/IGF-1R hybrids and/or IGF-1Rs. Additional studies are needed to further explore this possibility.

Negative actions of insulin on its gene expression and biosynthesis have also been investigated in rats [158,159] and β-cell lines [160]. Chronic infusion of insulin or the use of insulinoma-bearing rats (NEDH) resulted in a significant reduction in proinsulin production [158]. It is difficult to conclude from these studies that it is due to insulin, since these animals also had hypoglycemia. In the studies by Koranyi et al. (1992) however, hypeinsulinemic clamps were performed (insulin 4.1 mU/kg/min) at two different glucose concentrations, 3 and 8 mM [159]. Insulin infusion induced a significant reduction in insulin mRNA expression, at both glucose levels. However, Zhang et al. (1994) reported that long term incubation of HIT-T15 β-cells with high insulin in the presence of low (0.8 mM) or high (11.1 mM) glucose concentrations resulted in two outcomes. Namely, a significant decline in insulin mRNA expression was observed at low glucose concentration, whereas it was preserved at the high glucose concentration [160]. These studies indicate that the negative regulation by insulin of its gene expression might depend on local glucose concentrations. 

### 5.2. Negative Actions of Insulin on β-Cell Mass and Survival

Insulin and IGF-1 signaling through the IRS_2_/PKB(Akt) pathway plays a critical role in the maintenance of β-cell mass through both control of β-cell proliferation and inhibition of apoptosis. This was shown in experiments using global or β-cell specific IRS_2_ knockout mice [77,78,161], β-cell specific IR or IGF-1R knockout mice [75,101,120,140] or kinase-dead Akt transgenic mice [70,162]. These studies suggested that common molecular defects in insulin/IGF-1 signaling pathways may underlie both, peripheral and β-cell insulin-resistance, leading to β-cell decompensation and development of T2D. The role of hyperinsulinemia and prolonged exposure to high insulin in β-cell insulin resistance and decompensation is still unclear and widely debated. Some researchers [22,23], including our group [24], have suggested that in conditions of insulin resistance and hyperinsulinemia, prolonged exposure to high concentrations of insulin may induce insulin resistance in β-cells and negatively impact their function and mass. 

Hyperinsulinemia caused by ectopic transplantation of rat insulinoma cells was shown to induce a significant reduction in β-cell mass [163,164]. This reduction in β-cell mass was shown to be the result of increased β-cell apoptosis in these animals. In addition, prolonged exposure to high insulin primes apoptosis and ER-stress-inducing mechanisms and leads to a reduction in β-cell viability, due to increased oxidative stress in Min-6, RINm, INS-1 β-cell lines and isolated mouse and human islets [22,23]. These studies did not explore the effects of prolonged exposure to insulin on the insulin/IGF-1 signaling pathway in β-cells. We have data, using INS1E β-cells and isolated rat islets, which show that β-cells develop insulin, as well as IGF-1 resistance, when subjected to prolonged exposure to high insulin [24]. We show that phosphorylation of several downstream signaling molecules of the IRS_2_/AKt/P70S6K and the IRS_2_/Raf-1/Erk1/2 branches of the insulin/IGF-1 signaling pathway, in response to an acute 5 min stimulation by 10 nM insulin or 5 nM IGF-1, was downregulated following a 24 h pre-exposure to high insulin [24]. These chronic insulin-induced impairments in insulin/IGF-1 signaling were dose- and time-dependent and were associated with increased [Ca^2+^] i, increased β-cell apoptosis and increased ER-stress markers [24].

Our findings of disturbed intracellular Ca^2+^ signaling and increased [Ca^2+^] i following prolonged exposure to insulin confirm previously reported studies demonstrating that insulin modulates Ca^2+^ signaling in β-cells. These studies have shown that negative feedback actions by insulin on its own secretion involve a PI3K-dependent activation of KATP-channels, leading to cell membrane hyperpolarization, inhibition of Ca^2+^ efflux and inhibition of insulin secretion [150,151]. Sabatini et al. (2019), in a valuable review, discuss the good and bad actions of intracellular Ca^2+^-signaling in β-cell function and survival. A short term increase of [Ca^2+^] i in β-cells promotes insulin granule exocytosis and insulin secretion [165,166], but also stimulates a number of signaling cascades that mediate proliferation and survival of β-cells [166,167,168]. A long-term increase in [Ca^2+^] I, however, was shown to trigger ER-stress-mediated β-cell apoptosis and contribute to β-cell failure [169,170]. This is in agreement with our own findings that show that the chronic insulin-induced increase i n [Ca^2+^] i leads to increased ER-stress markers, i.e., eIF2α^S51^ phosphorylation, Bip (GRP78) expression and activation of caspase-12, a substrate for the calcium-sensitive proteases, calpains, known to promote ER-stress-mediated β-cell apoptosis [171,172]. 

### 5.3. Insulin and the Islet Microenvironment

β-cells reside in a complex islet microenvironment where they interact with other endocrine cells, such as glucagon-secreting β-cells, somatostatin-secreting β-cells, pancreatic polypeptide-secreting PP cells, ghrelin-secreting ε-cells, and with vascular endothelial cells, as well as neuronal projections. Differences in islet architecture between rodents and humans have been reported [173,174]. Rodent islets have a mantle-core structure, in that non-β-cells are mostly found in the periphery while β-cells are in the center of the islet [175,176]. Human islets, however, were shown to be very heterogenous with respect to size, cell composition and islet architecture, and they do not have the mantle-like organization reported in rodents [173]. Pancreatic islets are highly vascularized; the islet capillary network is approximately five times denser in islets than in the exocrine pancreas [177]. Real-time fluorescence imaging demonstrated that the islet capillary network in mice exhibits an inner-to-outer flow pattern, where capillaries perfuse β-cells before the other islet cells [178]. Islet capillaries are highly fenestrated, containing 10 times more fenestrae than the exocrine pancreas [177,179]. This allows an efficient direct communication between β-cells and the intra-islet capillaries, ensuring a rapid response to increases in blood glucose levels by secreting insulin [180,181]. 

Studies have reported a bidirectional interaction between islet β-cells and vascular endothelial cells (EC), in that EC-derived factors promote β-cell differentiation, survival and islet development, while β-cell-derived angiogenic factors promote EC recruitment and islet vascularization [182,183,184]. In addition, islet blood flow can be regulated by metabolic and nonmetabolic factors. For example, glucose was shown to double islet blood flow [185], while insulin decreases it [186]. This was suggested to likely be due to insulin-induced hypoglycemia rather than to the hyperinsulinemia per se. However, positive and negative actions of insulin on peripheral vasculature and on endothelial cells have been reported [187]. Hyperinsulinemia was suggested to exert deleterious effects on the endothelium and cause endothelial insulin resistance [188,189]. Studies have shown that in animal models of obesity and T2D, such as Zucker fatty (ZF) and Zucker diabetic fatty rats, islet capillaries initially expand during the obesity-induced increase in β-cell mass, then decrease as diabetes develops and animals present β-cell mass loss [190]. More interestingly, insulin-deficient mice have more islet capillaries and bigger islets [191], again suggesting that insulin inhibits islet vascularization.

## 6. Concluding Remarks

There is convincing evidence that insulin signaling is essential for β-cell function and mass. Positive and negative actions of insulin, particularly on insulin secretion, have been reported, raising concerns about the significance of these contradictory findings. We propose a unifying concept that might explain these discrepancies in that positive and negative actions of insulin may reflect two sides of the same coin. At short term, secreted insulin in response to elevated blood glucose (in an autocrine manner) acts to stimulate its own gene expression ensuring that β-cells have sufficient pre-proinsulin to accommodate insulin demand, but also inhibits it (via negative feedback) when local insulin concentration becomes high and glucose reaches a normal level. Under normal physiological conditions and in conditions of insulin resistance, insulin regulates β-cell mass through enhanced proliferation and reduced cell death by apoptosis. These positive effects of insulin ensure that β-cell function and mass are maintained. However, prolonged exposure to high insulin could be detrimental to islet and β-cell function by inducing islet and β-cell insulin resistance. The overlooked role of the IGF-1Rs and IR/IGF-1R hybrids in these negative actions by insulin may be critical to β-cell decompensation. We also should not forget that high insulin could negatively affect islet vasculature. This may contribute to the lack of key factors secreted by endothelial cells that are known to promote β-cell differentiation and preserved mass. Vice versa, high insulin-mediated β-cell insulin resistance may interfere with the ability of the β-cell to promote normal endothelial function and modulate islet capillary density and blood flow.

**Figure 2:**
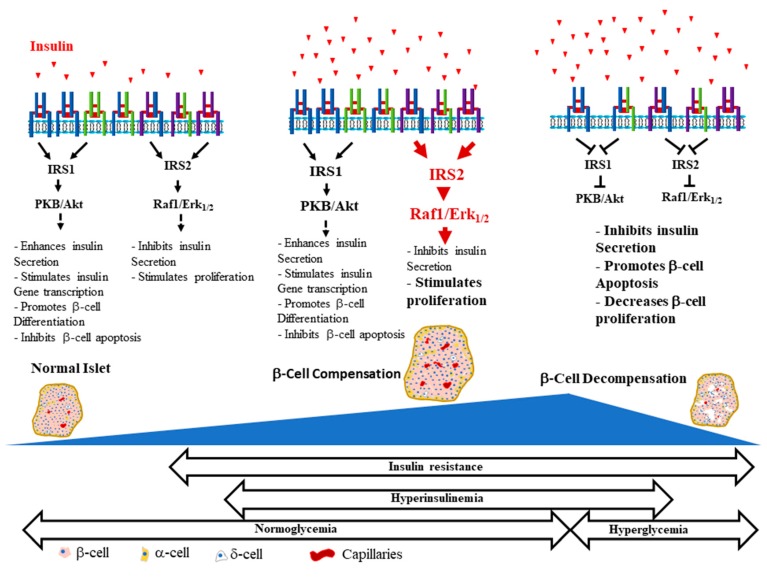
β-Cell life and death in the development of the obesity-mediated type 2 diabetes mellitus (T2DM). A simplified representation of the possible events involved in β-cell health and disease during the life time of a β-cell. The essential roles of insulin and IGF-1 signaling pathways in β-cell function and mass are depicted and the deleterious effects of their dysregulation are described. We included hyperinsulinemia, a key culprit factor that is often overlooked, which we believe might contribute to the common defects in the insulin and IGF-1 signaling pathways at the periphery and in islet β-cells that lead to the development of T2DM.

We strongly believe that integrated functions of all cell types, endocrine and non-endocrine, which form the islet are critical for normal islet and β-cell function. Hyperinsulinemia-mediated islet and β-cell insulin/IGF-1 resistance, often overlooked, could be a key factor that contributes to β-cell decompensation (Figure 2). This certainly will compromise the reciprocal communications between islet cells that are required for normal islet and β-cell function. Impairments of these communications, for example between β-cells and endothelial cells, may influence whether β-cells can adequately respond to insulin resistance and maintain normoglycemia. Studies have shown that early initiation of insulin therapy in patients with T2D helps preserve β-cell function, in addition to reducing cardiovascular complications and improving the quality of life [192,193]. One setback of early insulin therapy, however, is that it is associated with hyperphagia, weight gain and lipogenesis [194,195], which could exacerbate the insulin resistance and the metabolic alterations observed in T2D and lead to intensified treatment.
